# Early progression of pulmonary hypertension in the monocrotaline model in males is associated with increased lung permeability

**DOI:** 10.1186/s13293-020-00289-5

**Published:** 2020-03-18

**Authors:** Olga Rafikova, Joel James, Cody A. Eccles, Sergey Kurdyukov, Maki Niihori, Mathews Valuparampil Varghese, Ruslan Rafikov

**Affiliations:** Division of Endocrinology, Department of Medicine, University of Arizona, Tucson, AZ 85721 USA

**Keywords:** Pulmonary hypertension, Heme signaling, Lung permeability, Sex difference, Endothelial barrier function

## Abstract

**Background:**

The mechanisms involved in pulmonary hypertension (PH) development in patients and pre-clinical models are poorly understood. PH has a well-established sex dimorphism in patients with increased frequency of PH in females, and more severe disease with poor survival prognosis in males. Previously, we found that heme signaling plays an essential role in the development phase of the Sugen/Hypoxia (SU/Hx) model. This study is focused on the elucidation of sex differences in mechanisms of PH development related to heme action at the early stage of the monocrotaline (MCT) PH model.

**Methods:**

Rats received MCT injection (60 mg/kg, i.p.) and followed for 14 days to investigate early disease changes. Hemodynamic parameters were recorded at the end of the study; plasma, lung homogenates, and nuclear fractions were used for the evaluation of protein levels.

**Results:**

Our data indicate that on day 14, rats did not show any significant increase in the Fulton index due to the early disease phase. However, the right ventricular systolic pressure was significantly increased in male rats, while female rats showed only a trend. Interestingly, only males demonstrated an increased lung-to-bodyweight ratio that indicated lung edema. Indeed, lung histology confirmed severe perivascular edema in males. Previously, we have reported that the increased perivascular edema in SU/Hx model correlated with intravascular hemolysis and activated heme signaling. Here, we found that elevated free hemoglobin levels and perivascular edema were increased, specifically in males showing more rapid progress of PH. A high level of heme carrier protein 1 (HCP-1), which is involved in heme uptake from the bloodstream into the cells, was also found elevated in the lungs of males. The upregulation of heme oxygenase in males indicated increased intracellular heme catabolism. Increased heme signaling resulted in the activation of heme-mediated barrier-disruptive mechanisms. Thus, hemolysis in males can be responsible for increased permeability of the lungs and early disease development.

**Conclusions:**

Our study indicates the importance of barrier-disruptive mechanisms as an earlier event in the induction of pulmonary hypertension. Importantly, males are more susceptible to hemolysis and develop PH earlier than females.

## Background

Pulmonary hypertension (PH) affects vascular circulation in the lungs. Pulmonary vasculature becomes narrowed due to vasoconstriction, proliferation, fibrosis, or thrombosis, leading to increased pressure in the pulmonary circulation and increased workload for the right heart [1–3]. Patients with PH die due to the right heart failure. However, the main problem stems from vascular wall cells’ proliferation, which includes endothelial [4–6], smooth muscle cells [7, 8], and fibroblasts [9, 10]. There are several animal models available to study pulmonary hypertension, including the well-established monocrotaline (MCT) [11, 12] and Su5416/hypoxia (SU/Hx) [13, 14] models. The MCT model involves a one-time injection of the plant toxin, monocrotaline that metabolizes in the liver, and produces the monocrotaline pyrrole. MCT pyrrole induces endothelial damage via induction of oxidative stress. This oxidative stress occurs due to the binding of monocrotaline pyrrole to free cysteine residues or by direct inactivation of catalase [15]. SU/Hx model reproduces the main characteristics of human-like pulmonary hypertension with high pulmonary pressure, irreversible proliferative remodeling in the pulmonary artery with plexiform lesions formation, and right heart hypertrophy. However, the mechanism of pulmonary hypertension induction is still in debate. As a VEGFR2 blocker, SU5416 induces initial damage to the endothelial cells by preventing their growth and by induction of apoptosis [13, 14]. Thus, endothelial damage could be a common mechanism of disease initiation in both animal models.

Pulmonary hypertension is a complex disease, which includes activation and inhibition of the same pathways throughout the course of the disease. For example, apoptosis initially activated in PH is later replaced by the uncontrolled growth of the cells in the vascular wall [14]. Using the MCT model at the early stage of the disease, 14 days after MCT injection, we previously demonstrated that activation of many important pathways involved in PH pathobiology precedes any histological and pathophysiological changes [16]. Thus, we found that at the beginning of the disease, cells in the lungs significantly change their metabolism, leading to alterations in arginine/nitric oxide pathway, glycolysis, prostaglandin synthesis, glutathione balance, extracellular matrix formation, and activity of inflammatory pathways. Therefore, focusing on early events in the pulmonary circulation can shed light on important mechanisms leading to pathological changes in PH [2].

Recently, we have reported that patients with PH have increased levels of circulating free hemoglobin (Hb), a marker of hemolysis, which strongly correlated with the clinical characteristics of PH progression [17]. In the SU/Hx model, hemolysis was associated with an increased heme-mediated endothelial barrier dysfunction and formation of perivascular edema. These changes were especially evident in the early stages of the disease. Therefore, in the present work, we aimed to investigate the early stage of the MCT model for heme-mediated pathways as well as a possible contribution of sex, by separate analysis of male and female rats.

## Methods

### Animal model

A total of 24 Sprague Dawley rats (12 weeks old), 12 male and 12 female animals, were used in this study (*N* = 6 per group). A total of 6 male and 6 female animals were injected intraperitoneally (i.p.) with monocrotaline (MCT) at 60 mg/kg at day 1 of the experiment. Six control animals were injected i.p. with monocrotaline vehicle. Animals under study were housed at the University of Arizona’s Animal Care Facility until day 14 of the experiment.

Animals were housed at an ambient 22 °C with a 12-h light/dark cycle. All animals received standardized rodent food and water ad libitum. The handling procedures and experimental design were approved by the University of Arizona Institutional Animal Care and Use Committee.

### Hemodynamic measurements and sample collection

Fourteen days post-MCT/vehicle treatment; animals were anesthetized with Inactin (Sigma-Aldrich, T133) at the dosage of 100 mg/kg, i.p. A customized pressure transducer catheter (SPR-513, Millar Instruments, Houston, TX) was inserted through the right jugular vein and advanced into the right ventricle (RV) to monitor right ventricular systolic pressure (RVSP) and right ventricular diastolic pressure (RVDP) as described previously [17]. Briefly, the pressure transducer catheter was connected to a Millar Transducer Control Unit TC-510 and PL3504 PowerLab 4/35 data acquisition system (AD Instruments, Colorado Springs, CO) to continuously record RV pressure under small animal ventilator system (Harvard Rodent Ventilator-683; Harvard Apparatus, South Natick, MA). The thorax was opened, 4–5 mL of whole blood was collected into a heparinized syringe through the right ventricle (RV), centrifuged at 1000*g* for 2 min; separated plasma was aspirated and snap-frozen in liquid nitrogen for later analysis. Next, the lungs were flushed with 0.9% sodium chloride through the RV. Heart and lungs were subsequently collected from the animals; the RV free wall was separated from the left ventricle (LV) and the septum (S). The Fulton index (RV/LV + S ratio) was assessed as a parameter of RV hypertrophy. The total wet lung weight was measured and normalized to the bodyweight of the animal. The left lung was fixed in formalin and embedded in paraffin for histological examination. The right lung was snap-frozen in liquid nitrogen and stored at −80 °C for further biochemical studies.

### Cell-free hemoglobin measurement

Plasma analysis of cell-free hemoglobin was done utilizing gel electrophoresis and subsequent excitation at 488 nm, as described previously [17]. Briefly, 1 uL of plasma was diluted 10 times with PBS, solvated with 6× non-reducing Laemmli sample buffer (Boston Bioproducts Inc., Ashland, MA), loaded into 4–20% SDS-PAGE Mini-PROTEAN TGX™ gels (Bio-Rad Laboratories Inc., Hercules, CA) and separated by electrophoresis. The auto-fluorescent bands were visualized by 488 nm light emitted in a ChemiDoc™ MP Imaging System (Bio-Rad Laboratories Inc., Hercules, CA) and analyzed using Image Lab™ software.

### Western blot analysis

Total lung protein was analyzed as previously described [17]. Briefly, the frozen lung was lysed in a permeabilization buffer with protease and phosphatase inhibitor cocktail (ThermoScientific) and mechanically homogenized using a Fisher Homogenizer 850. The samples were centrifuged at 10,000*g* for 10 min, and the supernatant was collected. Protein quantification was done utilizing a BCA protein assay kit (Pierce™, Rockford, IL). A total of 20–40 μg of protein were incubated with 6× Laemmli sample buffer (Boston Bioproducts Inc., Ashland, MA) for 5 min at 95 °C, and loaded into 4–20% SDS-PAGE Mini-PROTEAN TGX Stain-Free™ gels (Bio-Rad Laboratories Inc., Hercules, CA) and separated by electrophoresis. Protein was transferred from the gel onto PVDF membranes using the Trans-Blot Turbo transferring system (Bio-Rad Laboratories Inc.) and blocked with 5% bovine serum albumin in Tris-buffered saline. Membranes were probed for HCP1 (B-4) (Santa Cruz, sc-393460), Heme Oxygenase 1 (A-3) (Santa Cruz, sc-136960), NFE2L2 (Cell Signaling, 12721S), NRF1 (Cell Signaling, 69432), Bach1 (Santa Cruz, sc-271211), HSP27 (G31) (Cell Signaling, 2402S), Rac1 (C-14) (Santa Cruz, sc-217), P-MYPT1 (Thr696) (Cell Signaling, 5163S), MYPT1 (Cell Signaling, 2634S), P-MLC2 (Ser19) (Cell Signaling, 3671P), MLC2 (Cell Signaling, 3672S), Claudin-1 (Abcam, Ab140349), Claudin-5 (Abcam, Ab15106), ZO-1 (Cell Signaling, 5406S). Some membranes were re-probed for multiple proteins; probed membranes were stripped at room temperature for 10 min with membrane stripper (ThermoFisher). The protein loading was normalized per total sample protein using stain-free gels, as previously described [17]. This normalization is equal to housekeeping genes normalization and rigorously evaluated by the Bio-Rad company (http://www.bio-rad.com/en-us/applications-technologies/stain-free-imaging-technology?ID=NZ0G1815) and by our lab in comparison with beta-actin normalization.

### Lung tissue subcellular fractionation

Subcellular protein fractionation was completed utilizing FractionPREP™ Cell Fractionation Kit (BioVision Inc., Milpitas, Ca). The frozen lung tissue was processed as per the manufacturer’s provided protocol. Subsequently, the nuclear fractions were quantified with the BCA protein assay kit (Pierce™, Rockford, IL) and subjected to western blot analysis. Proteins were normalized on a stain-free total protein signal in the nuclear fraction.

### Histological analysis

For the morphometric assessment of pulmonary vessels, 5-μm tissue sections were dewaxed and stained with hematoxylin and eosin (H&E) by HistoWiz Inc. (histowiz.com) using standard operating procedures and a fully automated workflow. The wall thickness of the pulmonary artery (PA) was measured using the publicly available software Fiji ImageJ (http://fiji.sc/Fiji; in the public domain) [18].

### Statistical analysis

The means ± SEM were calculated, and significance was determined by the two-way analysis of variance (ANOVA) or the unpaired *t* test. The factors considered in the two-way ANOVA was the sex of the animal group and MCT treatment. For two-way ANOVA, Sidak’s post hoc testing was also utilized. The unpaired *t* test was carried out between the controls of the respective groups—male control vs male MCT and female control vs female MCT. A value of *P* < 0.05 was considered significant. Statistical calculations were performed using the GraphPad Prism 8 software.

## Results

### Male rats are prone to early susceptibility of the disease

**Fig. 1:**
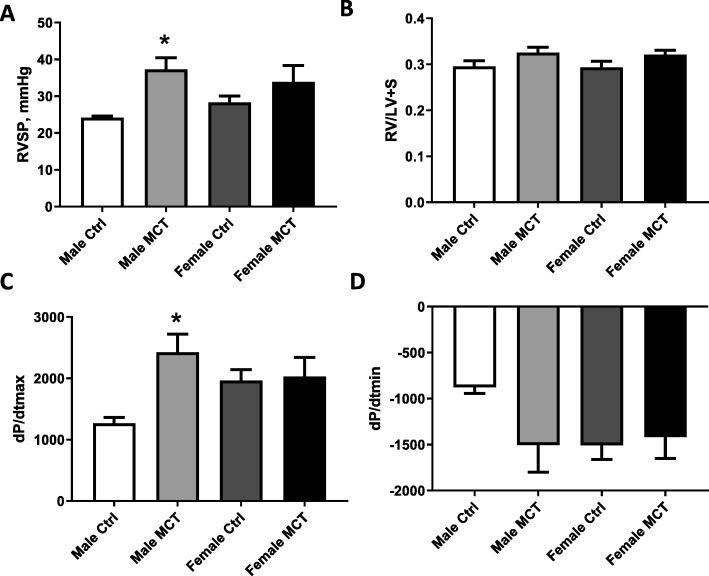
Hemodynamic changes in 14 days MCT model. Right ventricular (RV) systolic pressure found to be elevated significantly only in males treated with MCT. **a** Fulton index at this early stage of PH was unchanged between groups. **b** Contractility of the right heart (dP/dtmax) was significantly reduced in males, but not in females. **c** Relaxation parameter (dP/dtmin) did not reach statistical significance in males and was unaffected in females. **d** Mean ± SEM, *N* = 6, *p < 0.05 vs control, two-way ANOVA

In this study, the MCT rat model of PH was used. To assess the developmental phase of the disease as previously confirmed [16], the rats were analyzed 14 days after MCT injection. The male and female sexes were examined separately. Right ventricle catheterization indicates that at this early stage, the increase in the right ventricular systolic pressure (RVSP) was evident only in the male group (Fig. 1a). Right ventricle hypertrophy (measured by Fulton index) showed only a mild trend of increase in both males and females without reaching statistical significance (Fig. 1b). Still, the heart function in males was altered, as indicated by dP/dt values (Fig. 1c and d). Thus, there was a significant increase in RV contractility and a trend of increasing RV relaxation in males.

**Fig. 2:**
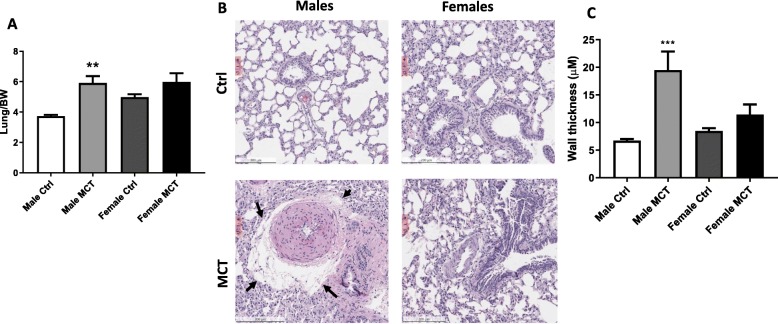
Lung edema in males treated with MCT. Lung weight normalized on the bodyweight showed a significant increase in males due to liquid accumulation in the lungs and indicated lung edema (**a**). Histology showed the formation of perivascular cuffs pronounced in male rats treated with monocrotaline (arrows) (**b**). Quantification of vessel wall thickness shows a significant increase in males (**c**) mean ± SEM, *N* = 6, **p < 0.01, ***p < 0.001 vs control, two-way ANOVA

We have previously found significant lung edema occurring at the early stages of the SU/Hx model. In this study, we observed a significantly increased lung weight in MCT-treated males (Fig. 2a), whereas female rats did not show increased lung weight. Histological examination of lungs revealed the formation of perivascular edema and increased pulmonary artery wall thickness in males (Fig. 2b and c) that was consistent with the lung weight findings and vascular remodeling. Indeed, we have recently shown a direct correlation between the manifestation of disease and lung edema in both MCT and SU/Hx models [2].

### Male rats demonstrate increased hemolysis in the early MCT model

**Fig. 3:**
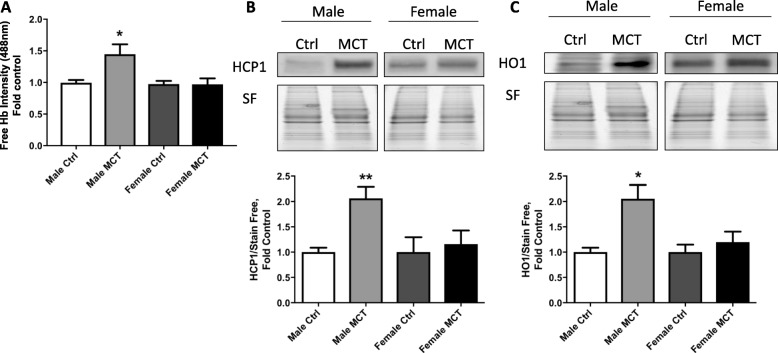
Hemolysis in early MCT model. Free hemoglobin signal in plasma showed a significant increase in male rats, but not female (**a**). Heme transporter, heme carrier protein 1 (HCP1), expression increased only in males (**b**). Inducible heme oxygenase (HO1) was activated in males (**c**). Mean ± SEM, *N* = 4–6, *p < 0.05,**p < 0.01 vs control, *t* test

Previously, we have reported that vascular permeability increases due to hemolysis, and free heme affect the endothelial barrier [17]. In order to evaluate hemolysis, we measured free hemoglobin levels in plasma. Our data indicate an increased level of free hemoglobin in males, whereas no changes were found in females (Fig. 3a). Heme uptake into vascular cells is regulated by heme carrier protein-1 (HCP1). We found that HCP-1 was upregulated only in males (Fig. 3b). Importantly, the heme oxygenase-1 (HO1), which is induced by increased heme concentration inside cells, was also found to be significantly upregulated in males (Fig. 3c). Thus, this confirms increased hemolysis and the entrance of free heme into the cells in males.

**Fig. 4:**
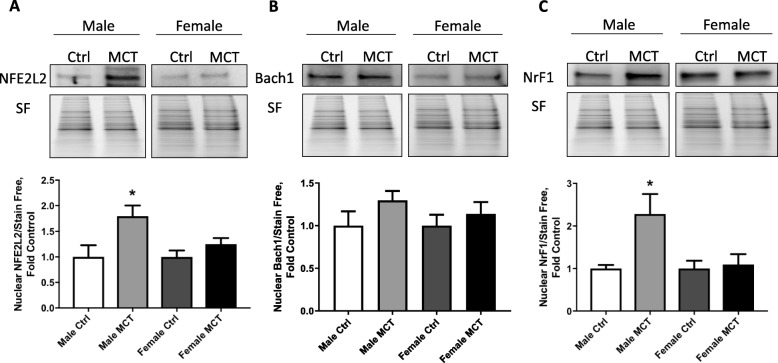
Translocation of transcriptional factors (TF). Nuclear fractions that were isolated from lung tissue were used to screen the activation of different transcription factors. It was found that the factor responsible for heme-mediated response, NFE2L2 (or NRF2), was activated in males only (**a**). Transcription suppressor BACH1 was unchanged (**b**). Energy regulating TF, NRF1, was also activated in males (**c**). Nuclear proteins were normalized over their respective total proteins (stain-free). Mean ± SEM, *N* = 4, *p < 0.05 vs control, *t* test

High levels of HO1 expression in males indicate a difference in transcriptional regulation between the sexes. To find out why males predominantly promote heme-mediated intracellular signaling, we tested the Nuclear factor erythroid 2-related factor 2 (NRF2) transcription factor (TF), which is linked with heme oxygenase and other antioxidant systems’ upregulation. NRF2 works together with a repressor, BTB Domain, and CNC Homolog 1 (BACH1). It has been previously shown that when heme binds to BACH1, it dissociates from the promoter region, which becomes available for NRF2 binding [19, 20]. Interestingly, we found that only males have an increased NRF2 expression that could be the primary mechanism of HO-1 upregulation (Fig. 4a) in males. The negative regulator of NRF2, BACH1, did not change in both males and females (Fig. 4b). Nuclear respiratory factor 1 (NRF1) is a TF, which upregulates in response to heme oxygenase and controls mitochondrial biogenesis. It was found to be increased in males (Fig. 4c). This result indicates that heme induces stress-related signaling and activates transcription factors only in males, leading to antioxidant response and mitochondrial biogenesis.

### Increased barrier-disruptive mechanisms in males in the early MCT model

**Fig. 5:**
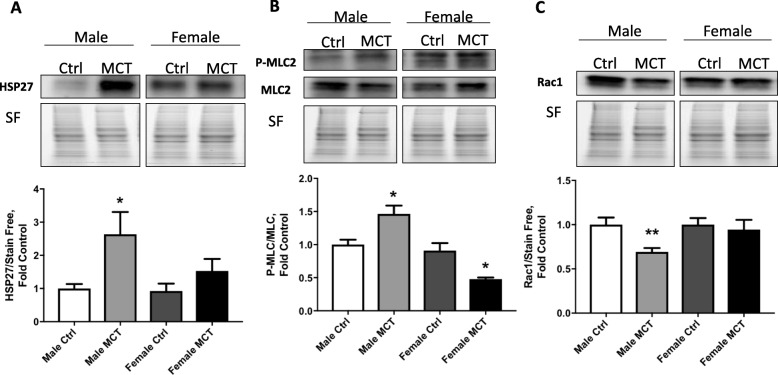
Heme-mediated actin remodeling mechanisms in the MCT model. Our data indicate that the level of HSP27 involved in actin fibers formation was highly elevated in the lungs of male rats after MCT treatment (**a**). This correlated with increased phosphorylation of MLC (**b**). Barrier protective small GTPase, Rac1, was significantly decreased in MCT males (**c**). Mean ± SEM, *N* = 4, *p < 0.05,**p < 0.01 vs control, *t* test

**Fig. 6:**
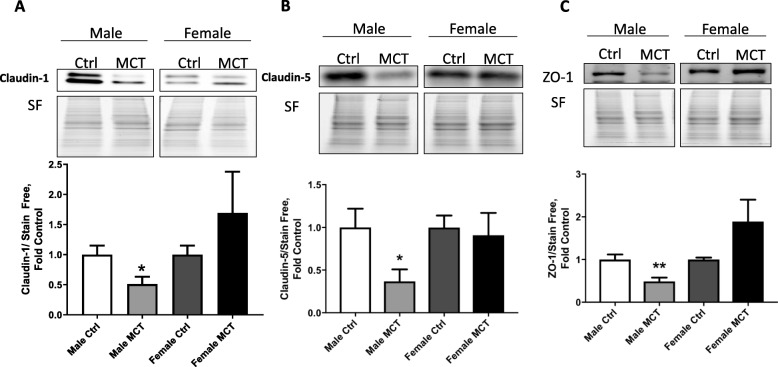
Heme-mediated effects on tight junctions. Proteins involved in the formation of tight junctions, claudins1/5, and ZO-1 were significantly reduced in MCT male rats (**a**, **b**, **c**). Mean ± SEM, *N* = 4, *p < 0.05,**p < 0.01 vs control, *t* test

To determine whether free heme is involved in increased vascular permeability, we tested proteins involved in endothelial cell-to-cell adherence and barrier function. We found that HSP27, a heat shock family protein that is responsible for stabilization of actin and formation of stress fibers, was upregulated specifically in males (Fig. 5a). Subsequently, activation of cytoskeleton remodeling by myosin light chain (MLC) phosphorylation was found to be significantly increased in males (Fig. 5b). Conversely, the barrier protective mechanism that manifests via the stabilization of cortical actin filaments by Rac1 small GTPase [21] exhibited attenuation in males (Fig. 5c). The redistribution of actin from the plasma membrane into stress fibers is usually accompanied by a rearrangement of tight junctions (TJ), leading to decreased adherence between endothelial cells. We examined the main TJ proteins, such as Claudin-1/5 and ZO-1. Our data indicate that TJ proteins were markedly reduced in males (Fig. 6a, b, c). This will result in the disruption of the endothelial barrier and fluid leakage from the bloodstream into the perivascular area, as we observed in male rats.

## Discussion

It is well known that the frequency of PH in hemolytic patients is extremely higher than in the general population [22–28]. We have previously reported a strong correlation between increased hemolysis and the worsening of hemodynamic parameters in PH patients [17]. On the molecular level, we found that heme-mediated signaling can contribute to PH development via activation of p38 MAPK signaling. However, the role of hemolysis in PH is not entirely understood. In this work, we attempted to investigate the role of sex in hemolysis-induced effects. Sexual dimorphism in PH is a well-known phenomenon [29]. Although female sex associated with increased risk of PH, males are having a severer disease and poorer prognosis. Our previous report in the Sugen/Hypoxia (Su/Hx) rat model showed that males develop inflammation and fibrosis in the lungs and heart, whereas females exhibited increased proliferation of pulmonary vasculature but adaptive right heart hypertrophy without fibrotic changes [30]. We also recently showed that rats with a mutation in NFU1 protein develop pulmonary arterial hypertension with a remarkable demonstration of sexual dimorphism as found in humans. Interestingly, in the NFU1 rat model, we found that the prevalence of the disease is more pronounced in female rats than in males [31]. In the present study, we found that the early MCT model indicates that males are more prone to develop hemolysis, and they showed increased pulmonary pressure and remodeling.

**Fig. 7:**
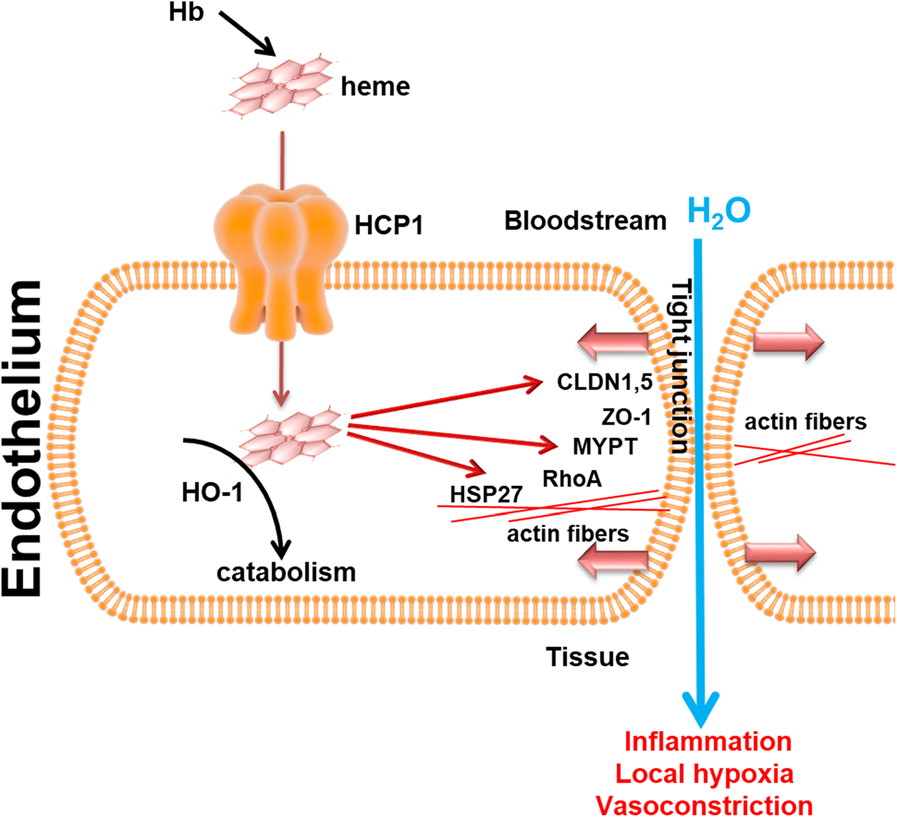
Overall mechanisms of heme-induced barrier dysfunction. During hemolysis, hemoglobin releases its co-factor, free heme. Through the heme carrier protein 1 (HCP1), free heme translocates into cells, leading to the activation of heme oxygenase and barrier disruptive mechanisms. Heme affects the barrier in two phases. First, heme induces actin fibers formation by upregulation of HSP27, inactivation of barrier protective MYPT and Rac1, leading to increased phosphorylation of MLC. The second phase is the dysregulation of tight junctions (TJ) between cells resulting in fluid leakage through the endothelial barrier. These effects of free heme contribute to the development of pulmonary hypertension via inflammation, vasoconstriction and local hypoxia due to the formation of perivascular cuffs

Next, in the MCT model, we also observed increased lung edema similar to previous reports in the Su/Hx model [17]. Therefore, lung edema could be linked to a hemolytic event in males. Upregulation of heme carrier protein-1 (HCP-1) found in the lungs of males results in increased free heme uptake. Thus, activating of heme influx will result in intracellular heme signaling only in males. This was confirmed by the activation of heme catabolism via upregulated heme oxygenase. We have also found that heme induces disruptive barrier mechanisms leading to a reduction in tight junction (TJ) proteins ZO-1, and claudins1,5, which regulates fluid retention in the bloodstream by the endothelial barrier [32, 33]. At the same time, we observed an increased actin stress-fibers formation signaling via HSP27 and Rac1 [34, 35]. All these events will ultimately lead to endothelial barrier leakage (Fig. 7). Importantly, those mechanisms were found to be upregulated in males only at the early stage of the disease.

One of the main questions of this study is how endothelial barrier dysfunction can contribute to the development of PH. Previously, we found that barrier dysfunction is pronounced during the early stages of pulmonary hypertension development [17]. Thus, this study was done on the 14 days MCT model, which is considered to be the initial stage. Leakage of pulmonary vasculature due to heme-mediated mechanisms results in fluid accumulation around the pulmonary arteries. This, in turn, can attract inflammatory cells and accumulate cytokines and chemokines. We have also shown a strong correlation between lung edema and pulmonary pressure, and the hypoxic environment of vessels surrounding by edema [2]. Moreover, this liquid layer will mechanically reduce the vasodilation of the arteries [36] and increase their stiffness. Finally, expansion of the perivascular area due to liquid accumulation will reduce gas and nutrients transport from vasa vasorum, leading to local ischemia. All those effects can induce initial damage to the pulmonary vasculature, induce hypoxia signaling leading to glycolytic switch and proliferation of smooth muscle cells and fibroblasts.

The predisposition of males to hemolysis and heme-activated pathways could be explained by the well-known observation that males have increased reactive oxygen species (ROS) generation, and the female hormone, estrogen, has an antioxidant action, protecting females [37]. Increased ROS generation was linked to the increased fragility of RBCs [38] that can contribute to elevated hemolysis in males. Moreover, our data indicate that males significantly upregulate heme oxygenase via NRF2 dependent mechanism. The role of heme oxygenase in PH is controversial [39–44]. On the one hand, heme oxygenase consumes the pro-oxidant, free heme, and produces an antioxidant, bilirubin. On another side, it releases carbon monoxide that can induce mitochondrial electron chain dysfunction and therefore contributes to a glycolytic switch. Moreover, heme oxygenase releases free iron, which is an essential oxidant involved in the Fenton reaction and is a cofactor of prolyl hydroxylases involved in the inactivation of HIF signaling. Thus, the overall vector of heme oxygenase action in PH is complex. Our data indicate that the activation of heme oxygenase in males is insufficient to stop heme-mediated signaling related to barrier disruptive mechanisms. Therefore, in spite of the activation of NFR2 and heme oxygenase, heme induces barrier leakage in the MCT model in male rats.

### Perspectives and significance

We found that elevated hemolysis in males results in a more active translocation of free heme into the cells due to an upregulation of heme carrier (HCP-1). Increased heme transport into the cell leads to the activation of heme oxygenase to catabolize intracellular free heme. Despite the heme oxygenase activation, intracellular free heme induces the multiple barrier-disruptive mechanisms via the reduction of tight junctions and activation of signaling proteins important for cytoskeleton rearrangement. The effects of free heme are illustrated in Fig. 7. This shows an important role of free heme signaling in barrier dysfunction at an early stage of PH. Therefore, treatments to reduce hemolysis and free heme should be applied for early-stage PH patients, patients with exercise-induced PH, and patients with familial PH, especially with the male sex.

## Data Availability

The datasets used and/or analyzed during the current study are available from the corresponding author on reasonable request.
